# Intracholecystic papillary neoplasm arising in a patient with pancreaticobiliary maljunction: a case report

**DOI:** 10.1186/s12957-020-02072-7

**Published:** 2020-11-09

**Authors:** Toshimitsu Iwasaki, Yasuhiro Otsuka, Yoichi Miyata, Takahiro Einama, Hironori Tsujimoto, Hideki Ueno, Sho Ogata, Yoji Kishi

**Affiliations:** 1 Department of Surgery, National Defense Medical College, 3-2 Namiki, Tokorozawa, Saitama, 359-8513 Japan; 2 Department of Pathology and Laboratory Medicine, National Defense Medical College, 3-2 Namiki, Tokorozawa, Saitama, 359-0042 Japan

**Keywords:** Intracholecystic papillary neoplasm (ICPN), Pancreaticobiliary maljunction, Pancreatobiliary reflux, Gallbladder cancer

## Abstract

**Background:**

Pancreaticobiliary maljunction (PBM) is a congenital abnormality in which the pancreatic and biliary ducts join anatomically outside the duodenal wall resulting in the regurgitation of pancreatic juice into the biliary tract (pancreatobiliary reflux). Persistent pancreatobiliary reflux causes injury to the epithelium of the biliary tract and promotes the risk of biliary cancer. Intracholecyctic papillary neoplasm (ICPN) has been highlighted in the context of a cholecystic counterpart of intraductal papillary mucinous neoplasm of the pancreas and the bile duct, but the tumorigenesis of ICPNs remains unclear.

**Case presentation:**

A 52-year-old Japanese woman was referred for the assessment of dilation of the bile duct. Computed tomography which revealed an enhanced mass in the gallbladder and endoscopic retrograde cholangiopancreatography confirmed that the confluence of the main pancreatic duct and extrahepatic bile duct (EHBD) was located outside the duodenal wall. Under the diagnosis of gallbladder cancer with PBM, cholecystectomy with full thickness dissection, EHBD resection, lymph node dissection, and hepaticojejunostomy were performed. Macroscopic examination of the resected specimen showed that the cystic duct was dilated and joined into the EHBD just above its confluence with the pancreatic duct, and the inflamed change of non-tumorous mucosa of gallbladder indicating that there was considerable mucosal injury due to pancreatobiliary reflux to the gallbladder. Histopathological examination revealed that the gallbladder tumor was a gastric-type ICPN with non-invasive component. Either *KRAS* gene mutation or p53 protein expression that were known to be associated with the carcinogenesis of biliary cancer under the condition of pancreatobiliary reflux was not detected in the tumor cells of ICPN.

**Conclusion:**

The present case might suggest that there was no association between PBM and ICPN. To reveal the tumorigenesis of ICPN and its attribution to pancreatobiliary reflux, however, further study is warranted.

## Background

Pancreaticobiliary maljunction (PBM) is a congenital abnormality in which the pancreatic and biliary ducts join anatomically outside the duodenal wall [1, 2]. This leads to the regurgitation of pancreatic juice into the biliary tract (pancreatobiliary reflux) and its pooling in the gallbladder and bile ducts. Persistent pancreatobiliary reflux causes injury to the epithelium of the biliary tract and promotes the development of biliary cancer [3–5].

Intracholecyctic papillary neoplasm (ICPN) is an exophytic tumor of the gallbladder consisting of dysplastic cells and occasionally associated with an invasive component. This neoplasm is considered as a cholecystic counterpart of intraductal papillary mucinous neoplasm (IPMN) of the pancreas and intraductal papillary mucinous neoplasm of the bile duct (IPNB) [6, 7]. ICPNs have been gaining attention, and the morphological definition has just been established only in recent years [6, 8], on the other hand, the tumorigenesis remains unclear.

We recently encountered a rare case of ICPN arising in a patient with PBM and could evaluate the association of pancreatobiliary reflux with the development of ICPN.

## Case presentation

**Fig. 1:**
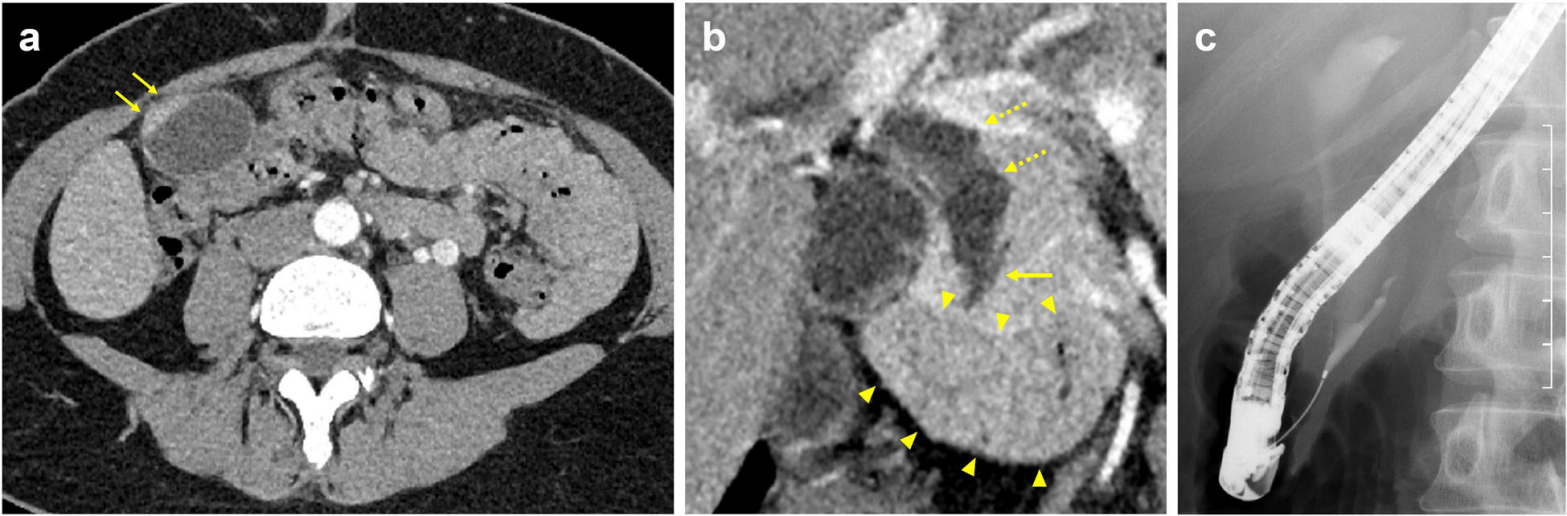
**a** Arterial-phase contrast computed tomography (CT) showing a mildly enhanced lesion in the gallbladder fundus (arrows). **b** Coronal image revealing that the dilated extrahepatic bile duct (EHBD) (dotted arrows) and the main pancreatic duct (arrow) joining into the EHBD at outside the duodenal wall (arrowheads). **c** Endoscopic retrograde cholangiopancreatography demonstrating the pancreaticobiliary maljunction

**Fig. 2:**
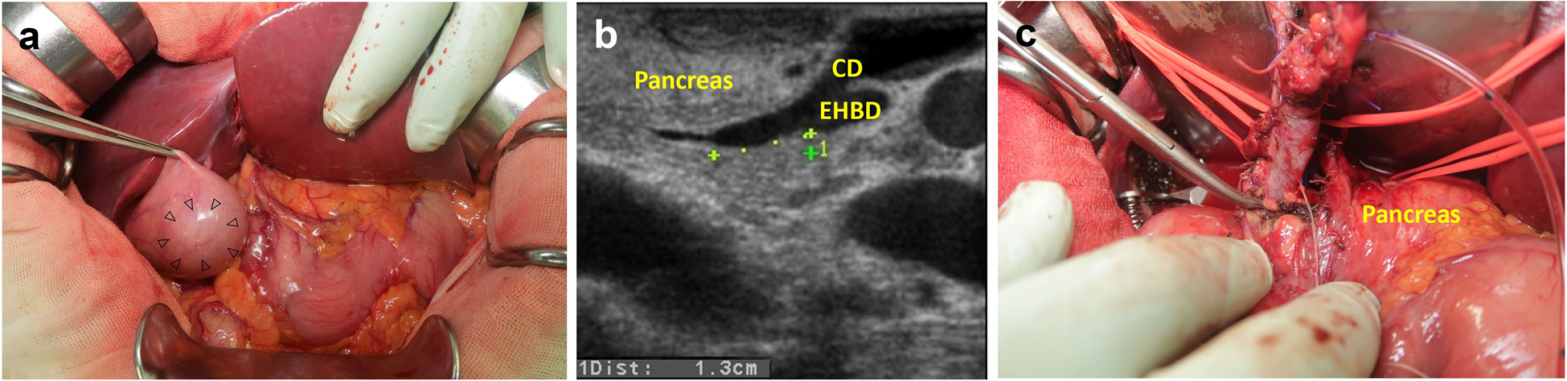
**a** Intraoperative findings suggesting no tumor invasion outside the gallbladder serosa. Arrowheads show the location of the tumor palpated at the peritoneal side of the gallbladder fundus. **b** Intraoperative ultrasonography showing the dilated cystic duct (CD) joining into the extrahepatic bile duct (EHBD) in the pancreas. **c** Intraoperative finding just the dissection at just above the confluence of main pancreatic duct and EHBD

A 52-year-old Japanese woman was referred to our hospital for the assessment of dilation of the bile duct detected at medical checkup detected by abdominal ultrasonography at a medical checkup. The patient was asymptomatic, and laboratory test results were as follows: serum total bilirubin, 0.61 mg/dL; aspartate aminotransferase, 16 IU/L; alanine aminotransferase, 15 IU/L; alkaline phosphatase, 164 IU/L; amylase (AMY), 72 IU/L; and carbohydrate antigen 19-9, 30 IU/mL (normal, ≤ 37 IU/mL). Contrast-enhanced computed tomography (CT) showed a well enhanced polypoid mass in the gallbladder (Fig. 1a). Dilation of the bile duct extended from the right and left hepatic duct to the level of the intrapancreatic bile duct. The confluence of the main pancreatic duct (MPD) and extrahepatic bile duct (EHBD) seemed to be located outside the duodenal wall, and the presence of PBM was suspected (Fig. 1b). Endoscopic retrograde cholangio-pancreatography (ERCP) confirmed that the PBM with a 10-mm-long common duct was above the ampulla (Fig. 1c). Cytology of the bile juice was negative for cancer. The AMY level of the bile juice sampled from the EHBD was 182,849 IU/L. Under the preoperative diagnosis of T1 gallbladder cancer with PBM of type IV-A according to Todani’s classification [9], surgical exploration was scheduled. On laparotomy, the tumor was identified by palpation at the peritoneal side of the gallbladder fundus. Macroscopically, there was no evidence of thickening or deformation of the gallbladder wall (Fig. 2a). Intraoperative ultrasonography revealed a papillary exophytic tumor at the peritoneal side of the gallbladder and confluence of the cystic duct (CD), and the EHBD was close to that of the MPD (Fig. 2b). From these findings, we confirmed that there was no tumor invasion to the gallbladder serosa or liver bed. Therefore, cholecystectomy with full-thickness dissection, EHBD resection, lymph node dissection, and hepaticojejunostomy was performed (Fig. 2c). Frozen section of the bile duct cut ends of hepatic and duodenal side showed negative for neoplasia.

**Fig. 3:**
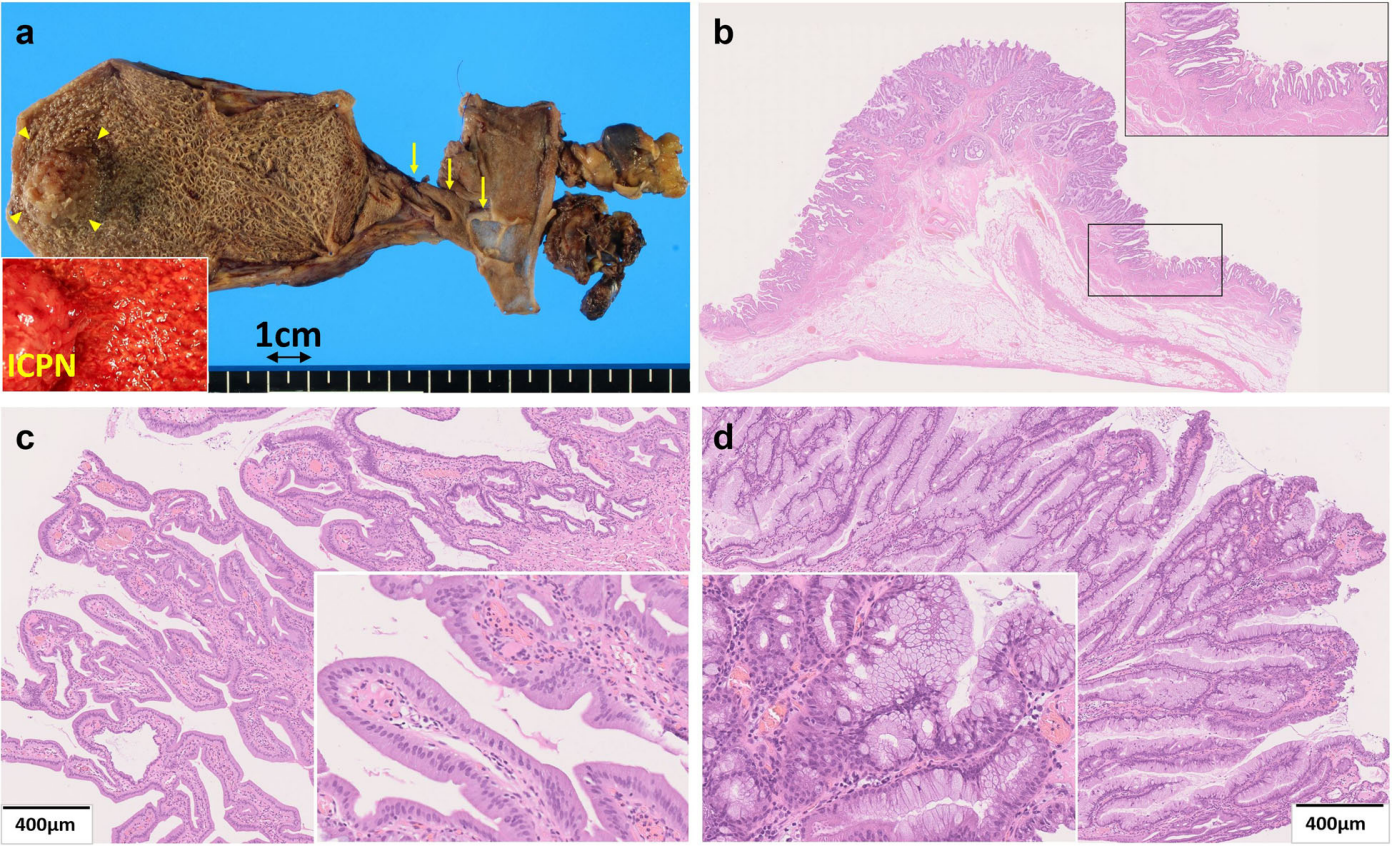
Gross pathologic findings and microscopic findings of the resected specimen. **a** Macroscopic findings of the formalin-fixed resected specimen. A cauliflower-like papillary tumor was located in the gallbladder fundus (arrowheads). The inset showed the macroscopic view of the fresh specimen showing the inflamed reddish hyperplastic mucosa of the gallbladder. The cystic duct was dilated (arrows). **b** Low-power view of the resected ICPN (hematoxylin and eosin [H & E]) showing the protruding tumor with a tubulopapillary architecture covered with neoplastic epithelium. The transient zone from the non-tumorous gallbladder to the ICPN was also covered with the same epithelium (inset). **c** Gastric pyloric component of the ICPN. Uniform, back-to-back mucinous grounds with features characteristic of pyloric glands. **d** Gastric foveolar component of the ICPN. Elongated, interconnecting tubules by tall columnar cells with abundant apical mucin with features characteristics of foveolar glands

**Fig. 4:**
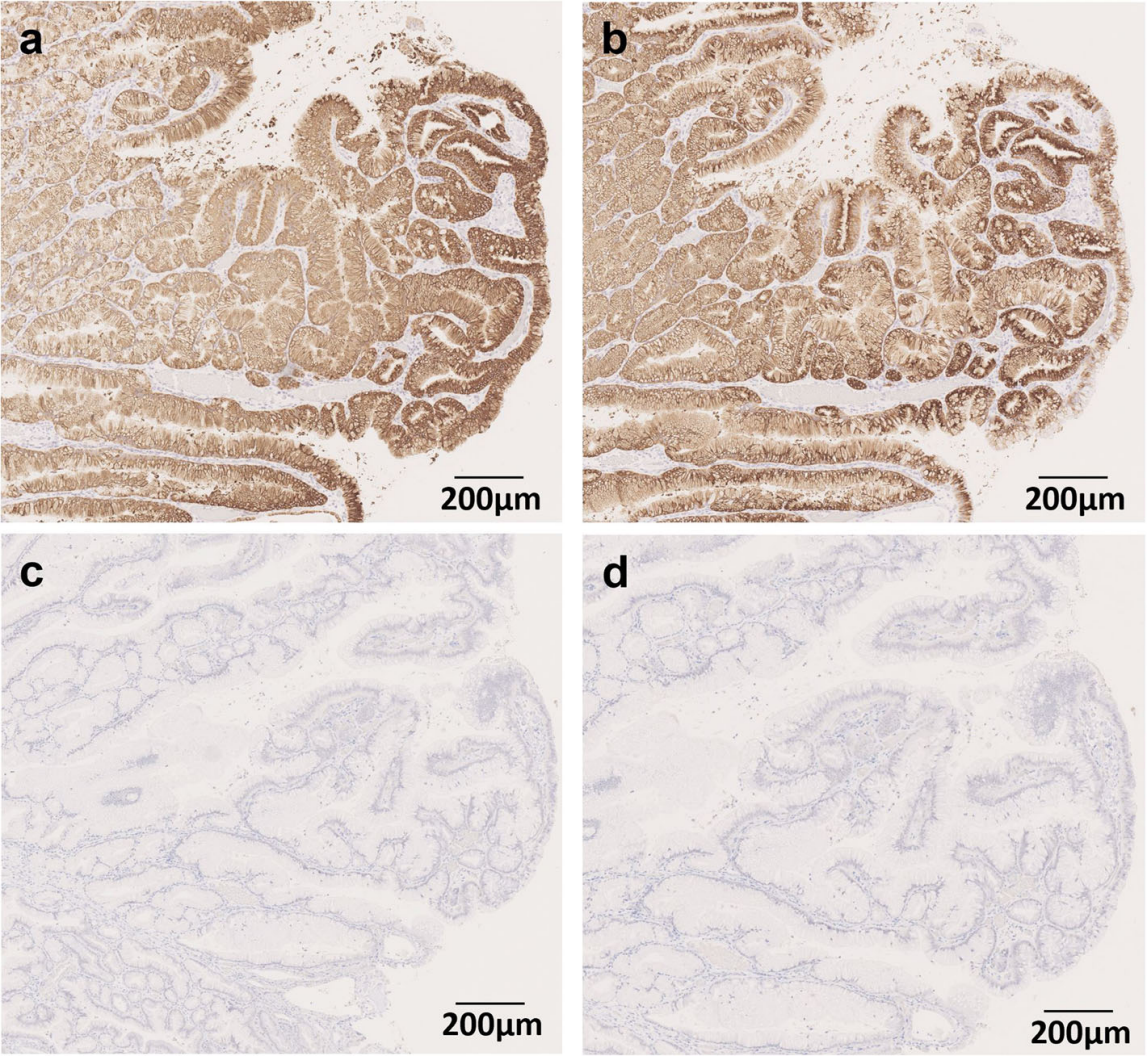
Immunohistochemical analysis of mucosal characteristics. **a** MUC5AC was positive. **b** MUC6 was positive. **c** p53 was negative. **d** β-catenin was negative

**Fig. 5:**
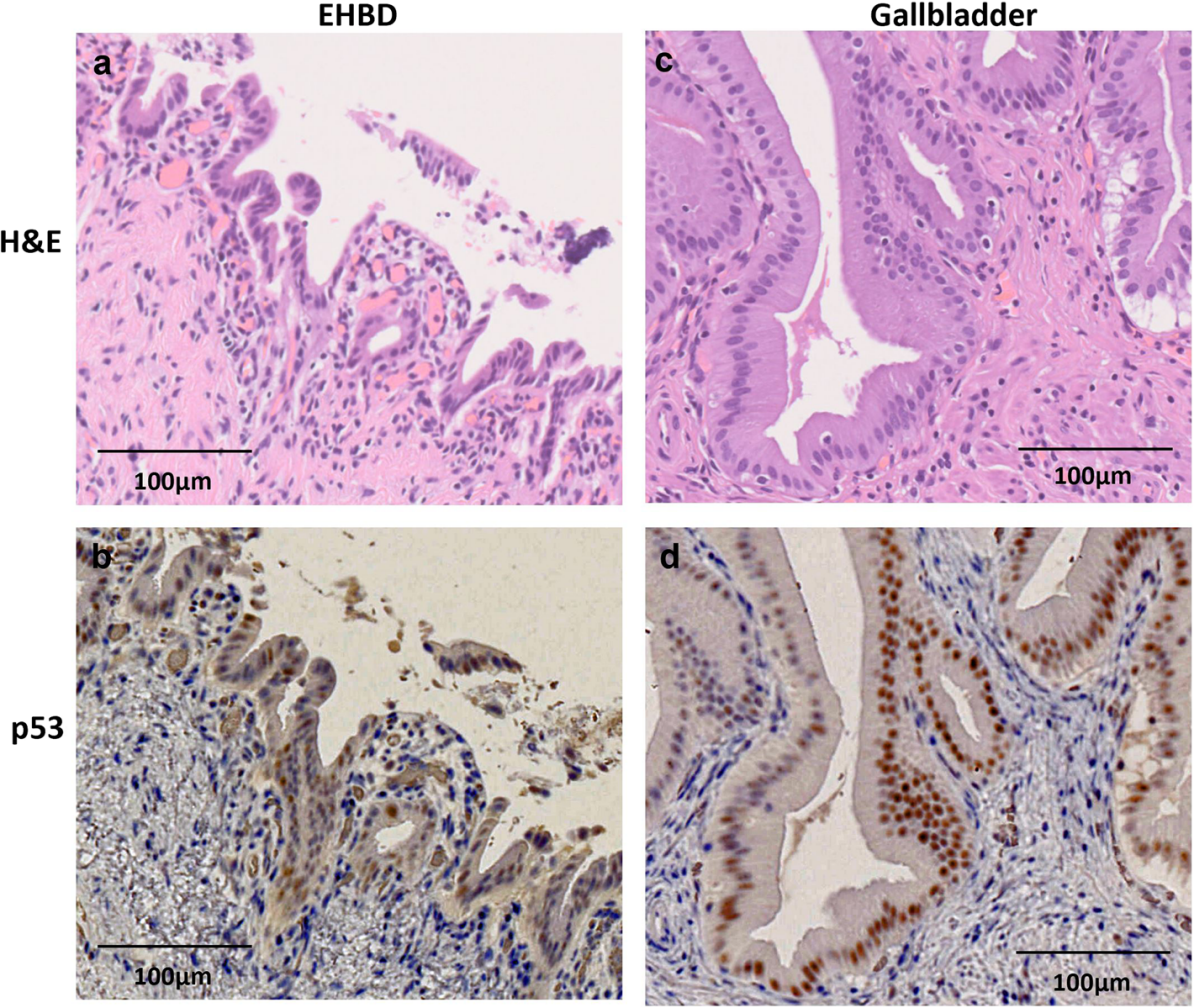
Immunohistochemical expression of p53 protein in the epitheliums of the extrahepatic bile duct (EHBD) (**a**, **b**) and the background mucosa of the gallbladder (**c**, **d**)

Gross examination of the resected specimens showed a polypoid tumor of 20 mm in size in the gallbladder fundus surrounded by the hyperplastic mucosa. The CD and the EHBD were dilated (Fig. 3a). There were no stones in the gallbladder, and no tumorous lesions in the CD or in the EHBD. Microscopic examination with the hematoxylin and eosin stain (Fig. 3b–d) showed that the gallbladder tumor demonstrated a tubulopapillary growth consisting of the tumor cells with gastric-type features including intracytoplasmic mucus, round nuclei, and clear cytoplasm. The tumor was located in the mucosa and had no invasive component. Immunohistochemistry (IHC) was performed using the following monoclonal antibodies: MUC1, MUC2, MUC5AC, MUC6 (Muc-1, Muc-2, Muc-5AC, Muc-6 glycoprotein, Novocastra), p53 (p53 Protein, DAKO), and β-catenin (beta-catenin, Novocastra). On immunostaining, the cells were diffusely positive for MUC5AC and MUC6 (Fig. 4a, b), focally positive for MUC2, and negative for MUC1. On the basis of the histologic findings and the 2010 World Health Organization (WHO) classification [7], the diagnosis of gastric-type ICPN was made. The surrounding hyperplastic epithelium of the gallbladder was also positive for MUC5AC and MUC6. The CD and EHBD were covered by epithelium with low-grade atypia. By referring to previous studies concerning the gene expression on the biliary tract epithelium exposed to the pancreatobiliary reflux as was summarized in Table 1 [8, 10–13], *KRAS* gene mutation and the expressions of p53 protein as well as β-catenin were assessed in the tissues from three sites as the follows: tumor cells of ICPN, background mucosa of the gallbladder surrounding the ICPN, and the epitheliums of EHBD. Analyses for gene mutations in codons 12/13, 59/51, 117, and 146 of the *KRAS* gene were performed by SRL, Inc. (Shinjuku, Japan), and IHC was performed to evaluate the expression of p53 protein and β-catenin (Fig. 4c, d). Neither *KRAS* gene mutation nor expression of β-catenin was detected in any of the three portions. On the other hand, p53 protein overexpression was detected in the epithelium of background gallbladder and EHBD but not in the ICPN (Figs. 4c and 5). The postoperative course was uneventful and the patient has been followed up without tumor recurrence for 5 months.

**Table 1:** Evaluation for the mutation and expression status of the epithelium exposed to the pancreatobiliary reflux

Site	*KRAS* gene mutation	p53 expression	β-Catenin expression
ICPN	(−)	(−)	(−)
Gallbladder	(−)	(+)	(−)
Extrahepatic bile duct	(−)	(+)	(−)

*ICPN* intracholecystic papillary neoplasm

## Discussion and conclusions

Premalignant changes are suggestive because they may hold clues to elucidate the carcinogenesis. In the 1990s, the concept of IPMN as a premalignant condition of pancreatic cancers was established [14], and later, IPNBs have been proposed as a biliary counterpart of IPMN [15, 16]. ICPN was described in the 2010 WHO classification [7] to designate the cholecystic counterpart. So far, the clinicopathologic features of ICPNs have been evaluated only in a few studies [6, 8, 17], and many issues especially concerning the tumorigenesis remain uncertain.

PBM is associated with the development of biliary tract cancer [18, 19]. Genetic mutation such as *KRAS* gene activation or the p53 tumor suppressor gene inactivation that occurs as a result of pancreatobiliary reflux is considered to play a role in carcinogenesis [4, 20]. Particularly, in our case, the CD joined into the EHBD just above its confluence with the pancreatic duct (Fig. 2b), and it was notable that not only the EHBD but also the CD was dilated. In addition, the inflamed change of background mucosa of the gallbladder was conspicuous (Fig. 3a). These findings indicated that there was a considerable mucosal injury by pancreatobiliary reflux not only to the EHBD but also to the gallbladder. We performed *KRAS* gene mutation analyses and IHC examination on p53 protein to evaluate whether the genetic alterations in the biliary tract, which had been affected by pancreatobiliary reflux, were also recognized in the ICPN. According to the recent study by Akita et al. [8] suggesting the specific contribution of the activated Wnt/β-catenin pathway in the tumorigenesis of ICPNs, expression of β-catenin was also evaluated (Table 1).

Overexpression of p53 protein was verified in the epithelium of the background gallbladder and EHBD, indicating that, in our case, pancreatobiliary reflux had caused epithelial injury and aberrant expression of p53 protein at the gallbladder mucosa and EHBD. On the other hand, *KRAS* gene mutation and expression of β-catenin were not detected in any of the examined tissue. In addition, none of the molecular abnormality examined in this work was not detected in the tumor cells of ICPN. These suggest that there might be no association between the pancreatobiliary reflux and the tumorigenesis of ICPN, and two possibilities are considered as to the development of the neoplasm. One is a silent mutation in the tumor suppressor gene p53. Another is the adaptive development regardless of the abnormality in the p53 signaling pathway, considering the concept that the tumorigenesis is an acquired adaptation for responding to a permanent regenerative signal in the context of tissue injury [21].

Besides the present case, only two cases of ICPN in the presence of PBM have been reported [8, 22]. According to Akita et al., there was no significant difference in the relationship with PBM between the three groups of patients with ICPN, papillary gallbladder cancer (GBC), and non-papillary GBC. In Akita et al.’s report, no concrete evidence suggesting PBM as a cause of ICPN was available [8]. In another case reported by Meguro et al. [22], the AMY level of the bile juice in the gallbladder was not elevated, possibly due to the dilution by mucin produced from the ICPN. Therefore, the contribution of pancreatobiliary reflux to the development of ICPN remains unclear. In this respect, the present report was the first to evaluate the association of pancreatobiliary reflux with the development of ICPN.

Previous studies reported the indolent nature of ICPNs. Adsay et al. reported that 1-, 3-, and 5-year survival rates of patients with non-invasive ICPNs were 90%, 90%, and 78%, respectively. In addition, even the patients with invasive carcinoma associate with ICPN showed better prognosis than those with conventional GBC (median survival, 35 months vs. 9 months) [6]. Akita et al. also reported that the survival rate of the patients with ICPN was better than either of those with non-papillary GBC or papillary GBC [8], suggesting that ICPN held different features from other papillary GBCs. Although some aspects of ICPNs are being elucidated, genetic features and the tumorigenesis of ICPNs remain unclear.

We experienced a rare case of ICPN arising from a gallbladder that had been exposed to pancreatic juice persistently as a result of PBM. Further study, which focused on the injury from pancreatobiliary reflux and on the adaptation to that, is warranted to reveal the tumorigenesis of ICPN.

## Data Availability

The datasets used and analyzed in this report are available from the corresponding author on reasonable request.

## Abbreviations

PBM: Pancreaticobiliary maljunction
ICPN: Intracholecystic papillary neoplasm
EHBD: Extrahepatic bile duct
IPMN: Intraductal papillary mucinous neoplasm
IPNB: Intraductal papillary mucinous neoplasm of the bile duct
AMY: Amylase
MPD: Main pancreatic duct
ERCP: Endoscopic retrograde cholangiopancreatography
CD: Cystic duct
IHC: Immunohistochemistry
WHO: World Health Organization
GBC: Gallbladder cancer
